# The Functional-Cognitive and Sensory Treatment (F-CaST) to improve rehabilitation outcomes of individuals with substance use disorder: a study protocol for a mixed-method randomized controlled trial

**DOI:** 10.1186/s13722-024-00449-7

**Published:** 2024-04-09

**Authors:** Naama Assayag, Tami Bar-Shalita, Debbie Rand

**Affiliations:** Department of Occupational Therapy, School of Health Professions, Faculty of Medical and Health Sciences, Tel Aviv University, 6997801 Tel Aviv, Israel

**Keywords:** Substance use disorder (SUD), Executive Functions, Sensory modulation dysfunction, Sensory processing, Goal achievement, Therapeutic community

## Abstract

**Background:**

Substance use disorder (SUD) is associated with executive function (EF) deficits and sensory modulation dysfunction (SMD). Yet, these deficits are not addressed therapeutically. This study aims to examine the effectiveness of the Functional-Cognitive and Sensory Treatment (F-CaST) compared to standard care to improve everyday performance and behavior and length of stay at the therapeutic community (TC) in individuals with SUD. In addition, to assess the improvement in EF, sensory modulation, participation, self-efficacy, life satisfaction, and use of strategies within and between groups. Satisfaction with F-CaST will also be assessed.

**Methods:**

Forty-eight participants from a community of men in a TC, aged 18–45 years will be randomly allocated to (i) F-CaST—(experimental group) providing sensory and EF strategies for improving daily function; (ii) standard care (control group) as provided in the TC. Assessments will be conducted by assessors blind to group allocation at 4 time points: T1- pre-intervention; T2- post-intervention; T3- 1-month follow-up; and T4- 3-month follow-up. Primary outcome measures will be everyday performance, assessed by the Canadian Occupational Performance Measure (COPM), behavior and length of stay in the TC; secondary outcome measures will assess EF, SMD. Semi-structured in-depth qualitative interviews will be conducted at T1, T2 and T4.

**Discussion:**

We hypothesize that F-CaST will lead to improved everyday performance and longer length of stay in the TC, compared to the control group. If F-CaST will prove to be effective, cognitive and sensory strategies may be incorporated as an adjunctive intervention in SUD rehabilitation.

*Trial registration*: ClinicalTrials.gov NCT05647863 Registered on 13 December 2022, https://classic.clinicaltrials.gov/ct2/show/NCT05647863.

## Background

Substance use disorder (SUD) is an urgent public health concern characterized by cognitive, behavioral, and physiological symptoms due to uncontrolled use of psychoactive substances despite harmful consequences. SUD severely impacts every life domain [1]. According to the National Survey on Drug Use and Health (2020), 58.7% of the US population aged 12 or older reported to use tobacco, alcohol, or an illicit drug in the past month. Of these, approximately 14.5% (40.3 million) are diagnosed with SUD [2], a two-fold increase compared to the previous year. In Israel rates are even higher, comprising 19.4% [3]. SUD has significant consequences across multiple life domains (e.g., work, school, home) and widespread ramifications in areas such as health (e.g. organ damage, infection, trauma or death) and society (crime, violence including domestic violence, and child abuse) [2]. It is an urgent public health concern with devastating effects that not only may persist across generations, but also cause a significant economic burden [4], estimated as more than $700 billion annually in the US [5].

Cumulative studies have demonstrated that SUD is associated with diverse cognitive impairments in attention, memory and executive functions (EF) [6, 7]. Importantly, although there are different profiles linked each to a specific type of substance, memory and EF are recognized as converging areas of deficit across different substance [6]. EF is an umbrella term comprising interrelated sets of abilities that direct and coordinate cognitive control. These are supported by dynamics of a superordinate, spatial distributed brain networks [8], allowing to perform rational decisions and emotion regulation, which contribute to efficient functioning and quality of life [9, 10]. The repeated use of substances leads to abnormal neural activity [11],related to learning and reward [12], and including the amygdala, hippocampus and prefrontal cortex. Indeed, a high prevalence of EF deficits have been reported to be present in SUD patients (53–70%) [13, 14], although it is controversial as to whether deficits in EF are a cause [15] or a consequence of SUD.

EF have been recognized as a key predictor for effective goal achievement and high self-efficacy, and therefore intact EF are crucial for treatment compliance and rehabilitation success [7, 16, 17]. Evidence clearly demonstrates that deficits in EF reduce successful SUD rehabilitation, specifically of cognitive behavioral therapies commonly provided in SUD rehabilitation programs [6, 13]. Indeed, dropouts, especially from long-term treatment frameworks, were recognized as a serious challenge in the treatment of SUD [13, 18]. Specifically, the estimated therapeutic community (TC) dropout rates stands up to 71% [18, 19], emphasizing that EF should be an important neurocognitive target in SUD interventions. Importantly, a recent systematic review concluded that cognitive remediation has the potential to pave the avenue for improving cognition and treatment outcomes in SUD [6].

Information processing that underlies EF is directly related to sensory perception. EF, specifically inhibitory control, plays an important role in the way sensory input from the environment is processed [20, 21]. Although deficits in EF have been found to be associated with difficulties in sensory processing within various populations across the life span (e.g., children with Autism Spectrum Disorders [21]; attention deficit hyperactivity disorder (ADHD); adults with specific learning disabilities [24] and older adults [25], there is limited research regarding populations with SUD. We have recently reported Sensory modulation dysfunction (SMD) in 54% of individuals with SUD (*vs.* 11.7% in a healthy comparison group) and revealed that sensory over-responsivity is a major contributing factor in SUD phenomenon [25].

SMD is a neuro-developmental condition, characterized by difficulty in regulating the degree, nature, or intensity of sensory stimulation in an adaptive manner [26, 27], consequently interfering with participation in everyday activities [28, 29] and quality of life [30–32]. The estimated prevalence of SMD is 5%-16% in the healthy general population [25, 33, 34]. Research studying the underlying mechanisms of SMD suggests alterations in neural processes [35–41] and anatomical abnormalities in sensory pathways [42] that may contribute to behavioral manifestations of sensory under- or over-responsivity. Sensory under-responsivity is manifested by disregarded or delayed responses to sensory stimulation, while sensory over-responsivity is characterized by experiencing non-painful sensations as irritating, unpleasant [26, 43] or painful [44].

Importantly, despite the fact that deficits in EF and SMD are common in SUD and have a substantial clinical relevance [45], as far as we know, there is no intervention combining both for individuals with SUD. Moreover, the majority of existing cognitive intervention programs used in SUD are restorative (or “bottom-up”) approaches [46], and their effectiveness have been tested using neuropsychological assessments and not by performance-based assessments [43, 45]. Our novel intervention, the Functional-Cognitive and Sensory Treatment (F-CaST) aims to bridge these gaps targeting the improvement of occupational performance, as has been recently suggested [48]. The F-CaST is an occupational therapy intervention aiming to provide cognitive and sensory strategies for improving daily functioning within the TC. The F-CaST teaches participants *“how to”* (vs. the more traditional virtue of “*what to*”). We based the F-CaST on the Functional and Cognitive Occupational Therapy (FaC_o_T) intervention, which was found to be effective in improving daily performance [49] and increasing self-efficacy [50] in adults with mild stroke. In addition, the F-CaST is based on The Ecological Model of SMD [51], which embraces the environment as a critical factor and recognizes the interplay between sensation, attention and emotion, which are linked to EF. Therefore, this proposed research aims to provide F-CaST, a novel intervention to improve rehabilitation outcomes for individuals with SUD living in a Therapeutic Community (TC).

We are planning to conduct a single-blind randomized-controlled clinical trial to determine whether the F-CaST is more effective than standard care for improving (1) everyday performance and (2) behavior and length of stay in the TC.

In addition, we aim to assess the improvement in EF, sensory modulation, participation, self-efficacy, life satisfaction, and use of strategies within each group and to compare between groups. For individuals in the F-CaST group, we aimed to evaluate their satisfaction with the intervention.

## Methods

**Fig. 1:**
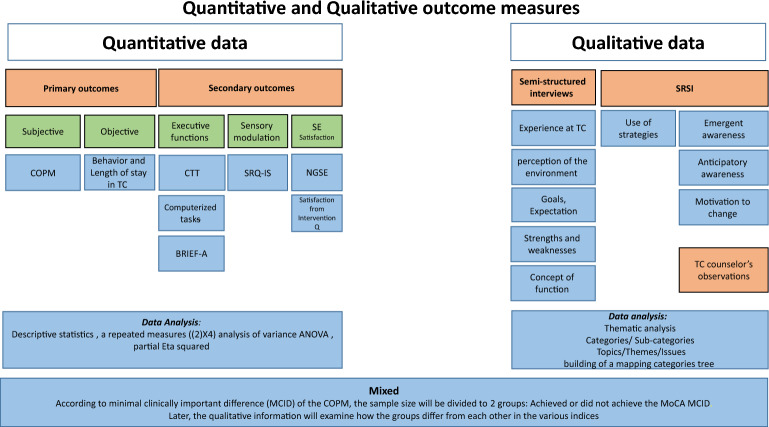
Mixed-methods approach; see the Quantitative and Qualitative outcome measures. This should appear below the Figure: T1- pre-intervention; T2- post-intervention; and T4- 3-month follow-up; COPM- Canadian Occupational Performance Measure; TC- Therapeutic Community; CTT- Color Trails Test; BRIEF-A- Behavior Rating Inventory of Executive Function-Adult Version; SRQ-IS-The Sensory Responsiveness Questionnaire-Intensity Scale; SE-Self Efficacy; NGSE- The New General Self-Efficacy Scale; SRSI- The Self-Regulation Skills Interview

A mixed-methods approach will be used; a single-blind randomized controlled trial comparing the F-CaST (experimental group) to standard care (control group) and a qualitative investigation (See Fig. 1).

**Fig. 2:**
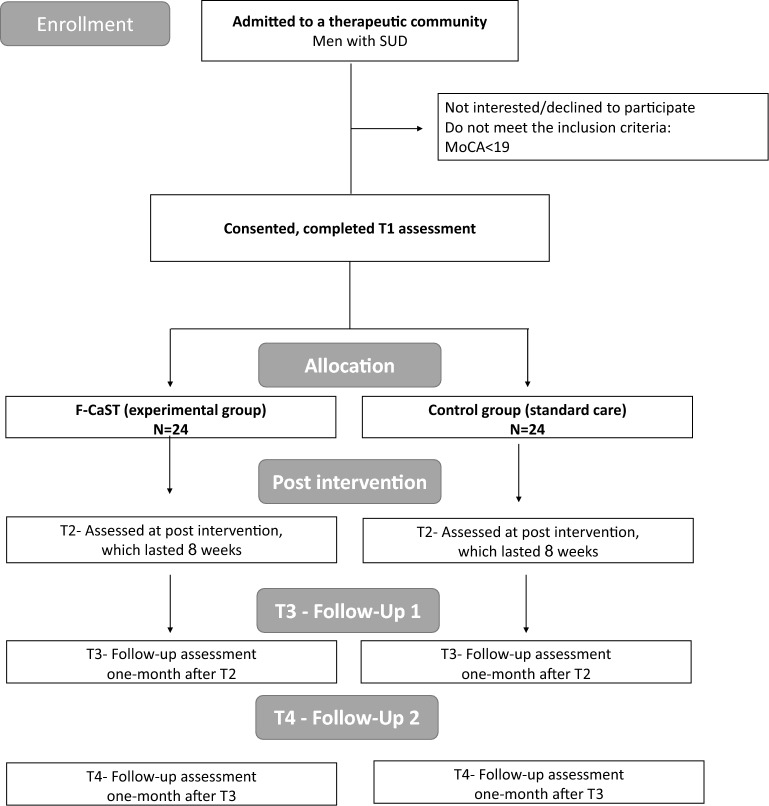
CONSORT Flow Diagram

Assessments will be conducted at four time points: T1 pre-intervention; T2 post-intervention; T3 one-month follow-up; and at T4 3-month follow-up, by assessors blinded to group allocation (See Fig. 2). Assessors are licensed occupational therapists, who are trained to administer and score all of the assessments. This study design will allow to identify changes in performance due to the intervention and verify whether the changes are stable. Since high dropout rates have been reported, repeated follow-up assessments are important. In addition, a qualitative investigation including in-depth interviews with the participants will be conducted to provide a deeper understanding of their perceived deficits and how these impact their everyday performance and treatment compliance in the TC (See Fig. 1).

Prior to the RCT we will conduct a prospective observational study with 4-point assessments including a group of individuals with SUD, receiving no additional treatment. This group of participants, although not randomized will form an additional control group.

## Setting

An adult community of men, within a TC. The TC is a rehabilitation center providing a controlled drug free environment with multidimensional support for individuals with severe SUD. Treatment in the TC is hierarchal, based on the duration and progress of the residents and usually lasts 1–1.5 years. The abstinence of the residents is routinely verified through random urine testing**.** Only individuals with the diagnosis of severe SUD (drug and/or alcohol use) and with no dual diagnosis are accepted into this TC. Diagnoses is done by a psychiatrist according to the Diagnostic and Statistical Manual of Mental Disorders-IV (DSM-IV).

## Participants

Forty-eight men with severe SUD residing in a TC will be recruited into the RCT. This study will recruit only men (a majority at the TC) and as an attempt to eliminate confounding factors. There is accumulating evidence of sex differences in SUD etiology and clinical manifestation (biological aspects, seeking treatment, consumption patterns) [52, 53].

### Inclusion criteria

Men will be eligible to take part if they meet the following inclusion criteria: Aged 18–45 years; Adequate language skills; Abstained from drugs and alcohol for at least 21 days (verifying minimum withdrawal effects); No more than 21 days of TC residency (ensuring acquaintance with the facility requirement and framework); With no dual diagnosis; Without a significant cognitive deficit [according to the Montreal Cognitive Assessment (MoCA) ≥ 19/30 points) a valid and reliable tool to assess cognition among patients with SUD [54, 55]]; Without other neurological conditions.

### Sample size

The sample size was calculated to (i) detect a minimally clinically important difference of the primary outcome measure of everyday (occupational) performance (COPM; see below) with 80% power and a significance level of 0.05, and (ii) to account for a 40% dropout. Thus 24 participants per group will be recruited providing a total of 48 participants for the study**.**

### Quantitative outcome measures

#### Primary outcomes measure

Every-day performance in the TC; Behavior and length of stay in the TC.

### Canadian Occupational Performance Measure (COPM) [56].

COPM is a semi-structured interview to capture the participant’s self-perception of everyday performance. Using 10-point scale participants are asked to identify and rate their performance and satisfaction from performance of three specific goals. The COPM has been found sensitive to change in the perceived occupational performance [57] and as a reliable and valid assessment for people with varied health conditions [58–60]. The COPM has been translated into Hebrew and used in other studies to assess daily performance e.g., [48, 49].

### Behavior and length of stay in TC

Information regarding participant behavior (discipline, number of times being late for scheduled activities) will be collected from the TC counselors. Length of stay (days) residing in TC. The Longer time spent in the TC, the better**.**

### Secondary outcome Measures

EF, sensory modulation, self-efficacy, life satisfaction, and use of strategies. We selected a variety of assessments to assure that we can capture many aspects of this novel intervention.

In-depth qualitative interviews will also be performed to provide additional insight related to the perceived link between EF, SMD and daily functioning in the TC.

### Executive functioning

Several tools will be used to assess EF in this population who may have  deficits in EF but are overall high functioning within the TC. EF in everyday situations via self-report will be assessed as well as neuropsychological tests specifically assessing planning, inhibition, and working memory.

### Color trails test (CTT) [60].

A pen and paper neuropsychological test used to measure cognitive flexibility, working memory and processing speed. The CTT includes two parts: (1) CTT 1 requires connecting a series of 25 numbered circles that are scattered on a sheet of paper and (2) CTT2 requires connecting numbered circles from 1 to 25 in sequence alternating between two colors—pink and yellow (e.g.,1-pink, 2-yellow, 3-pink, 4-yellow). The time (seconds) to complete each part is recorded. Less time indicates better performance. Completion time (up to 240 s) is translated to standardized score by normative data correcting for age and years of education. The CTT is widely used and is valid and reliable in a variety of populations [62].

### The Cambridge automatic neuropsychological test battery (CANTAB) [63, 64].

A computerized task (using a touchscreen tablet) will be used to assess multitasking. The **Multitasking Test (MTT) **[22] assesses participant’s ability to use multiple sources of potentially conflicting information to guide behavior. Arrows appear on the right or left side of the screen and participants receive instructions varying between "direction" and "side" for each trial. Some trials display congruent stimuli (e.g., arrow on the right side pointing to the right) whereas other trials display incongruent stimuli, which require a higher cognitive demand (e.g. arrow on the right side of the screen pointing to the left). The MTT was found with reasonable sensitivity (82.8%) and specificity (74.5%) for mild cognitive impairment [65]. The number of errors (Total incorrect), the median duration of the response time and the Multitasking Cost (difference between the median response time in the part where two rules are followed alternately and the one where one rule is followed) will be analyzed.

### Behavior rating inventory of executive function-adult version (BRIEF-A) self-report and informant report forms [66]

This 75-item questionnaire assesses EF in everyday situations referring to the past 30 days. The items graded on a 3-point scale: 'never', 'sometimes' or 'always' are summed-up into nine clinical scales which form two indices: the Behavioral Regulations Index (BRI), and the Metacognition Index (MI) and together produce an overall Global Executive Composite score (GEC). Raw scores are converted to T-scores where a score of 65 or higher denotes a clinical deficit. The three indices of the BRIEF-A have been shown with excellent internal consistency (Cronbach’s α: 0.93–0.94) and one-month test–retest reliability (r = 0.93–0.94), high ecological validity [66] and have been used as a sensitive measurement in patients with SUD [66].

### Sensory modulation

#### The Sensory Responsiveness Questionnaire-Intensity Scale (SRQ-IS) [68].

This standardized self-report questionnaire aims to clinically classify SMD in adults. Items represent typical daily life situations involving auditory, visual, gustatory, olfactory, vestibular and somatosensory sensations, excluding pain. Items are phrased either in a hedonic or aversive valence and are graded on a 5-point Likert scale: 'not at all' (1) to 'very much' (5) The SRQ provides two scores for each of the two SMD subtypes: SMD-SOR is determined by applying the SRQ-Aversive subscale score for scores higher than the normal cut-off score (mean + 2SD; 1.87 + 0.52); the SMD-SUR subtype is determined by applying the SRQ-Hedonic subscale score for scores higher than the normal cut-off score (mean + 2SD; 2.10 + 0.66). The SRQ been demonstrated to have test–retest reliability (r = 0.71–0.84; *p* < 0.001–0.005), internal consistency (Cronbach α = 0.90–0.93) and construct and criterion validity [68].

#### Self-efficacy

##### The New General Self-Efficacy Scale (NGSE) [69].

A self-report questionnaire to measure individual’s perceived capacity to achieve their goals despite their difficulties. Each of the eight statements (e.g. “Even when things are tough, I can perform quite well”) are rated using a 5-point rating scale: 'strongly disagree' [1] to 'strongly agree' (5). Higher scores indicate higher self-efficacy. The NGSE scale has high reliability and construct validity [68].

##### The Satisfaction from the intervention questionnaire [68, 71].

Following the F-CaST intervention, participants from the experimental group will be asked to rate their general and specific satisfaction from the intervention (e.g., How much did the intervention motivate you to make an effort?). Additional feedback will be also obtained (for example, was the intervention too long/just the right length/too short?). Each of the 9-items are rated separately. The questionnaire has been previously used to assess satisfaction in feasibility studies [70, 71].

##### Demographic questionnaire

Demographic data (age, years of education, employment status), information regarding substance consumption patterns [25] and health information will also be collected.

##### Qualitative data

Qualitative data will include a semi-structured interview and the use of strategies will be assessed.

#### Semi-structured interviews

Semi-structured interviews will provide a deeper understanding of the perspectives of the individuals with SUD and specifically to understand how adults with SUD perceive their everyday performance at the TC, the link between their performance to EF and to SMD deficits (See interview guide in Table 1). The interviews will be conducted pre and post-intervention (T1 and T2) and 12-weeks follow-up (T4). The interviews will be conducted by an  occupational therapist, not involved in the study, in a quiet room. The qualitative interviews will be audio-recorded, transcribed-verbatim and analyzed using content analysis.

**Table 1:** The semi-structured interview guide

Questions	Description
Experience in therapeutic community	Can you tell us about your experience in the TC? (Simplified question: How do you feel here? What is your opinion of this place and the people around you?)
Perception of the environment (physical and personal environment)	How do you feel within the TC? (To simplify or make it more detailed: For example, the people around you (counselors, other members) as far as the physical surroundings are concerned (the room, the building, dining room). If it is difficult to answer: Can you compare the TC to your previous living accommodations? How is it different? How does it make you feel?
Goals	What are the daily goals in your rehabilitation process? What challenges have you recognized in regards to your stay in the community? What will help you function in the community? (For instance, when you look ahead at your stay here, what do you think will be challenging?)
Strengths and weaknesses (events and coping mechanisms)	Tell me about an event or something that happened in the past week that you perceived as challenging. What happened? How did you deal with it?
Concept of function (supporting and restrictive factors in daily function)	As you approach the end of your stay, please tell me what in your functioning or your surroundings helped you remain in the community? Were there any occurrences that caused you to think about staying or leaving? Please give me an example What do you think would help you stay in the community going forward? Please relate to all the factors surrounding you in the community. What could possibly delay or cause your leaving the community?

##### The Self-Regulation Skills Interview (SRSI)[71].

A semi-structured interview to evaluate meta-cognitive abilities using a difficulty identified by the participant. Individuals are asked six questions relating to the identified difficulty. Emergent awareness: “Can you tell me how you know that you experience [the difficulty]?” Anticipatory awareness: “When are you most likely to experience [the difficulty], or in what situations does it mainly occur?”; Motivation to change: “How motivated are you to learn some different strategies to help overcome [the difficulty]?” (A self-rating between 0 and 10 was obtained); Strategy generation: “Have you thought of any strategies that you could use to help cope with [the difficulty]?”; Strategy selection: “What strategies are you currently using to cope with [the difficulty]?”; Effectiveness of strategies: “How well do the strategies that you are using work for you?” Each question is rated using a 10-point scale (0 = very high to 5 = moderate to 10 = very low) where scores reflect the level of awareness, motivation, strategy knowledge, or use of strategies. An average score is obtained, lower scores indicate better use of strategies. The SRSI has established interrater reliability (0.83 < α < 0.92); test–retest reliability (0.69 < α < 0.91) and concurrent validity [71].

## The interventions

### The F-CaST

The Experimental group will receive F-CaST, which focuses on teaching and practicing the use of strategies to compensate for deficits in EF and sensory modulation deficits to improve their daily performance. Participants will be provided with knowledge regarding their EF and sensory modulation difficulties in order to increase their awareness and understanding of how these deficits impact their daily function.

The F-CaST is based on the Functional and Cognitive Occupational Therapy (FaCoT) intervention which was developed for individuals with mild stroke, experiencing deficits in EF and emotional-behavioral symptoms [49]. The FaCoT was based on theoretical models from the field of cognitive rehabilitation and Bandura’s social learning theory. The aspects of FaCoT of teaching and practicing the use of cognitive and behavioral strategies to improve daily functioning and participation, were integrated into the F-CaST. The integration of the SMD field (based on the Ecological Model of SMD [51]), is novel, creating the F-CaST intervention. See Table 2 for additional details regarding the individual and group sessions.

**Table 2:** Description of F-CaST intervention

Content	Activities to facilitate learning
*Group sessions*	
Psychoeducational knowledge about the following topics which are then implemented in the individual sessions: - SUD and the impact on the brain - Reframing and conceptualizing function within the TC - Executive functions - Sensory modulation - The importance of executive functions and sensory modulation for efficient daily functioning in the TC - Strategies to overcome executive function deficits and for sensory modulation difficulties Sharing everyday situations according to the terms discussed The relation between Self-regulation and executive functioning in daily function	Group activities to encourage introspection to daily events through functional conceptualization Games and video clips that simulate different experiences that require executive functions or sensory modulation. Game-like activities to experience the effective use of strategies (in general) and specific executive functions or sensory strategies to overcome deficits Following these activities, sharing, reflecting on each self and others and group discussions will take place
*Individual sessions*	
The Psychoeducational knowledge that will be taught in the group sessions will be implemented to help each participant set their specific goals	Activity task analysis will be conducted regarding daily occupational goals that will be set by the participants For the first goal, both OT and participant will analyze and identify the specific difficulty within that goal Then an appropriate cognitive strategy will be selected to compensate for their EF deficits and promote their occupational performance. In addition, sensory strategies will be taught and integrated Cognitive strategies: Response inhibition, Initiation, Planning and Decision-Making Sensory strategies: Adjusting arousal levels and Self-regulation in the TC environment Behavioral strategies Self-perception, Situation interpretation and Future prediction will be taught and practiced using a positive persona (with high self-efficacy) and a negative persona (with low self-efficacy) in different everyday scenarios chosen as similar to each participant's goals After 3–4 sessions, the same process is done for another occupational goal and another cognitive strategy will be taught and practiced along the sensory and behavioral strategies

## Standard care

In the TC, there is a supervised and structured daily schedule in an environment free of substances. The standard care in the TC includes daily individual and group therapy sessions led by social workers and counselors. In addition, individuals have leisure and enrichment activities, etc.

### Procedure

Individuals with SUD consecutively admitted to the TC will be approached and invited to participate in the study. All assessments, will be administered by assessors blind to group allocation to minimize bias. Assessments will be performed in two sessions within one week and administration sequence will be counter balanced in two different orders to eliminate fatigue and attention span bias.

## Randomization

Following the T1 assessment participants will be stratified according to yes/no Attention deficit hyperactivity disorder (ADHD) based on The Adult ADHD Self-Report Scale-Version 1.1 (ASRS-V1.1)[73], a valid and reliable [74, 75] screening tool for ADHD. ADHD is prevalent in SUD population [76] and might affect intervention compliance and therefore will be controlled. Thereafter participants will be randomly allocated (using a simple block random allocation software) into the two arms. Allocation (1:1 ratio) will be concealed. Participants will be informed regarding their allocation by the research assistant who will not take part in the assessments or intervention.

## Data analysis

Descriptive statistics will be used to describe the participants in each group, in terms of their, demographic information and SUD parameters, primary and secondary outcome measures. Pre-intervention differences between the two groups will be analyzed using t-Test for independent samples (continuous measures) or Chi square (dichotomous measures). A repeated measures ((2)X4) analysis of variance ANOVA will be used to compare within- and between- group scores, for time (T1, T2, T3, T4) comparing the F-CaST to standard care and for interaction time*group effect.

To assess the clinical meaningfulness of the outcomes, the Partial Eta squared in ANOVA effect size will be calculated. Partial Eta squared is interpreted as the proportion of the total variability in the dependent variable that is accounted for by variation in the independent variable. ANOVA Partial Eta squared of 0.01, 0.06 and 0.14 are considered small, medium and large effect sizes (respectively).

Statistical significance will be set at p < 0.05, and all analyses will be conducted using SPSS for Windows version 27.0 (SPSS Inc, Chicago, IL). The transcripts of each of the four in-depth interviews will be analyzed using content analysis (an initial review, the development of mapping categories, and the building of a mapping categories tree) [77].

## Discussion

The aim of this mixed-method single-blind randomized controlled trial is to examine the efficacy of the Functional Cognitive and Sensory Treatment (F-CaST), an innovative personalized therapy in men with SUD residing in a TC. We expect that the F-CaST will lead to improved everyday performance and longer length of stay in the TC, compared to the control group. If the F-CaST will show to be effective, cognitive and sensory strategies may be incorporated as an adjunctive intervention in SUD rehabilitation and will support the need to provide intervention programs based on occupational performance to SUD population. Moreover, we believe that the effectiveness of the intervention program will be found at each of the assessments, thus strengthening the effectiveness of the intervention program over time.

The following study limitations will need to be taken into account. The fact that only men with SUD will be recruited from a single TC, will limit the generalizability of the findings to women. Future studies utilizing the F-CaST should definitely include women preferably from therapeutic communities. F-CaST group will be compared to a control group, who will receive standard care, and not an active control group. The duration of the intervention is short and therefore we might not be able to demonstrate effectiveness.

We envision that F-CaST, which is a novel intervention, will show to improve rehabilitation outcomes of men with SUD. F-CaST will fill gaps in current rehabilitation and expand our.

## Data Availability

Study data will be made available from the corresponding author upon reasonable request.
